# Early Outcomes of Three Total Arch Replacement Strategies for DeBakey Type I Aortic Dissection

**DOI:** 10.3389/fcvm.2021.638420

**Published:** 2021-04-15

**Authors:** Enzehua Xie, Jinlin Wu, Juntao Qiu, Lu Dai, Jiawei Qiu, Qipeng Luo, Wenxiang Jiang, Fangfang Cao, Rui Zhao, Shuya Fan, Wei Gao, Hongwei Guo, Xiaogang Sun, Cuntao Yu

**Affiliations:** ^1^Department of Cardiovascular Surgery, Fuwai Hospital, National Center for Cardiovascular Diseases, Chinese Academy of Medical Sciences and Peking Union Medical College, Beijing, China; ^2^Department of Cardiac Surgery, Guangdong Cardiovascular Institute, Guangdong Provincial People's Hospital, Guangdong Academy of Medical Sciences, Guangzhou, China; ^3^Department of Anesthesiology, Fuwai Hospital, National Center for Cardiovascular Diseases, Chinese Academy of Medical Sciences and Peking Union Medical College, Beijing, China

**Keywords:** DeBakey type I aortic dissection, aortic arch, total arch replacement, matching weight method, aortic balloon occlusion technique

## Abstract

**Background:** This study employed three surgical techniques: total arch replacement (TAR) with frozen elephant trunk (FET), aortic balloon occlusion technique (ABO) and hybrid aortic arch repair (HAR) on patients with type I aortic dissection in Fuwai Hospital, aiming to compare the early outcomes of these surgical armamentariums.

**Methods:** From January 2016 to December 2018, an overall 633 patients (431 of TAR+FET, 122 of HAR, and 80 of ABO) with type I aortic dissection were included in the study. Thirty-day mortality, stroke, paraplegia, re-exploration for bleeding, and renal replacement therapy were compared using the matching weight method (MWM).

**Results:** After MWM process, the baseline characteristics were comparable among three TAR groups. It showed that ABO group had the longest cardiopulmonary bypass (*p* < 0.001) and aortic cross-clamp time (*p* < 0.001), while the operation time was longest in the HAR group (*p* = 0.039). There was no significant difference in 30-day mortality among groups (*p* = 0.783). Furthermore, the incidence of stroke (*p* = 0.679), paraplegia (*p* = 0.104), re-exploration for bleeding (*p* = 0.313), and CRRT (*p* = 0.834) demonstrated no significant difference. Of note, no significant differences were found regarding these outcomes even before using MWM.

**Conclusions:** Based on the early outcomes, the three TAR approaches were equally applicable to type I aortic dissection. We may choose the specific procedure relatively flexibly according to patient status and surgeon's expertise. Importantly, long-term investigations are warranted to determine whether above approaches remain to be of equivalent efficacy and safety.

## Introduction

DeBakey type I dissection is a catastrophic emergency with high mortality and morbidity. A robust body of evidence have confirmed the efficacy and safety of the total arch replacement with frozen elephant trunk (TAR + FET) (1–4). However, there are certain risks associated with the use of hypothermic circulatory arrest (HCA) (5). In recent years, hybrid TAR (HAR) and aortic balloon occlusion (ABO) have also been gaining the popularity in our center, which would help to avoid or reduce HCA time. The HAR obviates the need for the circulatory arrest and the ABO technique can shorten the circulatory arrest time significantly.

As we continued to diversify out surgical armamentarium, it remains unknown the best techniques. In the current study, we aimed to compare the early outcomes of the aforementioned three strategies (TAR+FET, HAR, and ABO) to generate some evidence that may ultimately standardize or justify our clinical practice.

## Methods

### Study Population

Between January 2016 and December 2018, a total of 633 patients with acute DeBakey type I dissection were enrolled in this study. Patients were diagnosed with computed tomography. Transthoracic echocardiography or intraoperative transesophageal echocardiography was deployed to assess the morphology of the aortic valves. All patients underwent total arch replacement. And they were divided into 3 groups according to the surgical techniques. Four hundred thirty-one patients underwent TAR+FET; 122 patients underwent HAR; 80 patients underwent the ABO technique. Four fixed surgeons performed type I aortic dissection surgery in our center. All they were chief surgeon, had more than 15 years clinical experience, had performed more than 50 cases aortic operations, had the same operation experience. This study was approved by the institutional review board (Ethics Committee of Fuwai Hospital), with informed consent not required due to its observational nature. (Approval NO: 2017-877).

### Definitions

The postoperative complications and comorbidities were defined according to The Society of Thoracic Surgeons definitions (http://www.sts.org/national-database).

Penn classification was based on ischemic profiles (6, 7). Early mortality referred to death within 30 days after the surgery (including intraoperative death).

### Surgical Techniques

#### TAR+FET Group

The detailed procedures were described previously (8). In brief, median sternotomy was made and total cardiopulmonary bypass (CPB) was established. Arterial cannulation was performed at the right axillary artery in our daily practice. When CPB was established, cooling was also initiated to reach 32°C. When the cardiac arrest was achieved, the distal ascending aorta was clamped. During the cooling phase, the ascending aorta was managed according to the specific situation. When the nasopharyngeal temperature reached 20–25°C, the deep hypothermic circulatory arrest (DHCA) was instituted. Then antegrade cerebral perfusion (ACP) (flow rate: 8–12 mL/kg/min) was initiated. After 3 brachiocephalic vessels were clamped separately, we transected the aortic arch between the left common carotid artery and left subclavian artery. Under the direct vision, the FET stent-graft (MicroPort Medical Co., Ltd, Shanghai, China) was placed into the true lumen of the descending aorta. Then it was anastomosed to the 4-branched graft (Meadox Hemashield Platinum 4 Branch Graft, Boston Scientific Inc., Marlborough, Mass) in an end-to-end fashion. When distal anastomosis was completed, lower body perfusion was resumed. The left common carotid artery was anastomosed to the 4-branched graft, followed by rewarming. Then the proximal end of the 4-branched graft was sutured to the ascending aorta. Finally, the left subclavian and the innominate artery were sutured to the 4-branched graft in an end-to-end fashion.

### HAR Group (Hybrid TAR Without Circulatory Arrest)

All patients underwent type II hybrid aortic arch repair without circulatory arrest. Patients underwent a median sternotomy and the right femoral artery was cannulated. After the CPB and cooling phase (28–32°C), we repaired the aortic root or ascending aorta. Then, the proximal end of the 4-branched graft was sutured to the ascending aorta. The distal end of it was sutured to the position before the origin of the innominate artery, following which the rewarming initiated. Then, an end-to-end anastomosis was made between the 4-branched graft and the left common carotid artery, as well as the left subclavian artery and the innominate artery. The Z0 zone was converted into an artificial blood vessel. The lesions in the aortic arch were isolated by the stented graft that was anchored to the artificial blood vessel. Herein, we used four types of stents: Zenith (Cook Medical Inc., Bloomington, IN, USA), Relay (Bolton Medical, Sunrise, FL, USA), Talent and Valiant (Medtronic Inc., Santa Rosa, CA, USA), and Hercules (MicroPort Medical Co., Ltd, Shanghai, China). During the procedure, the surgeon should ascertain the patency of the left subclavian artificial vessel origin. Primarily, the stented graft was placed via femoral access. The surgical procedure was performed as one-stage procedure in the hybrid operating room and the operation time included the time for inserting a stent graft.

### ABO Group

It is different from the hybrid technique. The surgical procedures were carried out, as described previously (9). The ABO technique is an improvement to the TAR+FET as it decreases the circulatory arrest time. Patients underwent median sternotomy and CPB; when the nasopharyngeal temperature reached 28°C, the circulatory arrest was initiated. The right axillary artery was utilized to establish the ACP. After the aortic arch was transected between the left subclavian and the left common carotid arteries, the stented elephant trunk was implanted into the true lumen of the descending aorta (Cronus, MicroPort Endovascular Shanghai Co., Ltd, China). Concurrently, the aortic balloon with the sheath was placed into the metal region of the stented graft, followed by 40–45 mL normal saline injected into the balloon. The sheath could press the inflated balloon to a fixed position, following which, the perfusion of the lower extremity was restored via the right femoral artery, and the circulatory arrest time was reduced to ~5 min. Then, the balloon and sheath were removed when the 4-branched prosthetic graft was anastomosed to the descending aorta. After the left common carotid artery was reconstructed, the rewarming was begun, and CPB flow was returned to normal. The ascending aorta, the left subclavian, and innominate arteries were reconstructed sequentially.

Schematic diagrams of the three total arch replacement strategies are shown in the Supplementary Material 1.

### Statistical Analysis

Kolmogorov–Smirnov tests were performed to detect the normal distribution of continuous variables, which were presented as mean and standard deviation. For variables with a normal distribution and homogeneity of variance, a one-way analysis of variance (ANOVA) test was performed. The Kruskal-Wallis rank sum test was used for variables with a non-normal distribution. The categorical variables were described as frequency and percentage (%). Compared by the Chi-square test or Fisher's exact test. The matching weight method was used to calculate the weights. The matching weight method is an extension of inverse probability of treatment weighting (IPTW) that reweights both exposed and unexposed groups to emulate a propensity score matched population (10). The TriMatch package in R was used to compare the 3 groups. The balance between the 3 groups was assessed using standardized mean differences (SMD). After that, logistic regression was performed to assess the relationship between surgical strategies and 30-day mortality, with HAR group set as the reference.

A *P*-value threshold of 0.05 was considered significant. Statistical analyses were performed using R version 3.6.3.

## Results

### Patient Demographics

Patient characteristics were shown in Table 1. Between January 2016 and December 2018, a total of 633 patients [540 (85.3%) acute, 67 (10.6%) sub-acute, and 26 (4.1%) chronic] with type I aortic dissection were included in the study. We found the degree of urgency of the treated cases (122 HAR:105 (86.1%) acute,14 (11.5%) sub-acute, 3 (2.5%) chronic; 431 TAR+FET:368 (85.4%) acute, 45 (10.4%) sub-acute, 18 (4.2%) chronic; 80 ABO: 67 (83.8%) acute, 8 (10.0%) sub-acute, 5 (6.2%) chronic) (*p* = 0.761) was not significantly different between 3 groups. Most of the patients (85.3%) were acute type I aortic dissection. The proportion of patients with sub-acute or chronic type I aortic dissection is very small. They are not sufficient to affect the outcome. As for the time from referral to surgery, most of the patients underwent the surgery on the day of admission.

**Table 1:** Preoperative characteristics of the total cohort and each group before using the matching weight method.

	**Overall**	**HAR**	**TAR+FET**	**ABO**	***P*-value**
**Characteristic**	**(*n =* 633)**	**(*n =* 122)**	**(*n =* 431)**	**(*n =* 80)**	
Male (%)	460 (72.7)	77 (63.1)	332 (77.0)	51 (63.7)	0.002
Age	51.3 ± 11.8	61.6 ± 6.8	47.9 ± 11.0	53.9 ± 12.4	<0.001
BMI	26.3 ± 4.5	25.9 ± 4.0	26.5 ± 4.6	25.6 ± 4.1	0.125
Hypertension (%)	534 (84.4)	109 (89.3)	357 (82.8)	68 (85.0)	0.214
Diabetes mellitus (%)	24 (3.8)	7 (5.7)	11 (2.6)	6 (7.5)	0.047
CAD (%)	24 (3.8)	9 (7.4)	14 (3.2)	1 (1.2)	0.048
COPD (%)	7 (1.1)	3 (2.5)	4 (0.9)	0 (0.0)	0.216
MFS (%)	41 (6.5)	2 (1.6)	37 (8.6)	2 (2.5)	0.007
Smoking	259 (40.9)	42 (34.4)	188 (43.6)	29 (36.2)	0.126
Family history of aortic dissection (%)	5 (0.8)	0 (0.0)	5 (1.2)	0 (0.0)	0.307
History of cardiac surgery (%)	27 (4.3)	3 (2.5)	16 (3.7)	8 (10.0)	0.021
History of aortic surgery (%)	29 (4.6)	4 (3.3)	18 (4.2)	7 (8.8)	0.148
HB	136.5 ± 16.6	132.7 ± 16.4	137.3 ± 16.6	138.1 ± 16.4	0.018
WBC	11.6 ± 5.3	10.8 ± 3.9	11.9 ± 5.8	11.7 ± 4.3	0.127
PLT	195.9 ± 75.1	186.0 ± 74.3	198.1 ± 76.7	199.3 ± 67.2	0.264
Penn classification (%)					0.171
Penn Class a	492 (77.7)	103 (84.4)	328 (76.1)	61 (76.2)	
Penn Class b	123 (19.4)	18 (14.8)	86 (20.0)	19 (23.8)	
Penn Class c	14 (2.2)	1 (0.8)	13 (3.0)	0 (0.0)	
Penn Class b & c	4 (0.6)	0 (0.0)	4 (0.9)	0 (0.0)	

*TAR+FET, total arch replacement with frozen elephant trunk; ABO, aortic balloon occlusion; HAR, hybrid aortic arch repair; BMI, body mass index; MFS, Marfan syndrome; COPD, chronic obstructive pulmonary disease; CAD, coronary artery disease*.

Before MWM, 72.7% of the patients were men, and the average age of all patients was 51.3 ± 11.8 years. The patients in the HAR group was significantly older than the patients in the TAR+FET and ABO groups (*p* < 0.001). The proportion of patients with a history of cardiac surgery was higher in the ABO group (*p* = 0.021). The percentages of patients with Marfan syndrome (*p* = 0.007) was higher in the TAR+FET group. Patients in the HAR group had a lower hemoglobin level than the other two groups (*p* = 0.018).

**Figure 1:**
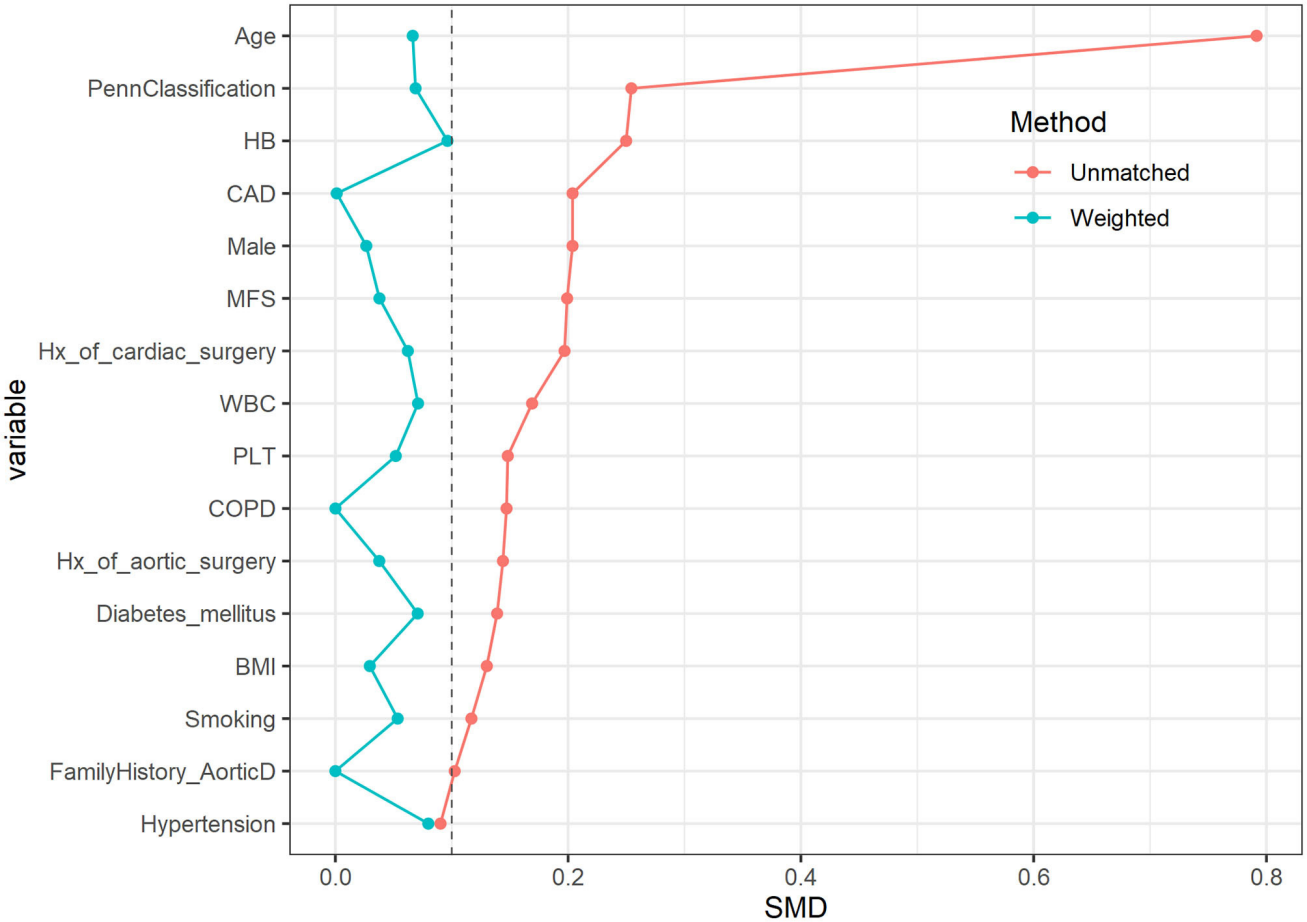
The love plot of standardized differences before and after matching. After using the matching weight method, the 3 groups were well-matched at baseline with the SMD of each variable < 0.1.

After MWM, there was no significant differences for the demographic characteristics (Table 2). The love plot shows that the 3 groups were well-matched at baseline with the SMD of each variable < 0.1 (Figure 1).

**Table 2:** Preoperative characteristics of the total cohort and each group after using the matching weight method.

	**Overall**	**HAR**	**TAR+FET**	**ABO**	***P*-value**
**Characteristic**	**(*n =* 169)**	**(*n =* 56.1)**	**(*n =* 56.1)**	**(*n =* 56.9)**	
Male (%)	103.1 (61.0)	33.7 (60.1)	34.8 (62.1)	34.6 (60.8)	0.949
Age	57.70 ± 9.41	58.20 ± 7.06	57.30 ± 9.80	57.59 ± 10.97	0.644
BMI	25.70 ± 3.92	25.63 ± 3.59	25.66 ± 3.90	25.81 ± 4.28	0.960
Hypertension (%)	148.6 (87.9)	50.2 (89.5)	49.7 (88.7)	48.7 (85.5)	0.618
Diabetes mellitus (%)	8.8 (5.2)	3.5 (6.2)	3.1 (5.6)	2.2 (3.8)	0.748
CAD (%)	3.0 (1.8)	1.0 (1.7)	1.0 (1.8)	1.0 (1.8)	0.999
COPD (%)	0.0 (0.0)	0.0 (0.0)	0.0 (0.0)	0.0 (0.0)	0.181
MFS (%)	2.1 (1.2)	0.7 (1.2)	0.9 (1.6)	0.6 (1.0)	0.813
Smoking	58.5 (34.6)	18.9 (33.8)	20.7 (36.9)	18.8 (33.1)	0.805
Family history of aortic dissection (%)	0.0 (0.0)	0.0 (0.0)	0.0 (0.0)	0.0 (0.0)	1.000
History of cardiac surgery (%)	8.8 (5.2)	2.4 (4.3)	2.7 (4.8)	3.7 (6.5)	0.795
History of aortic surgery (%)	7.2 (4.2)	2.0 (3.6)	2.4 (4.4)	2.7 (4.7)	0.893
HB	136.21 ± 16.78	135.07 ± 17.40	136.04 ± 15.89	137.51 ± 17.08	0.702
WBC	11.47 ± 4.34	11.60 ± 4.42	11.17 ± 4.10	11.63 ± 4.51	0.623
PLT	193.90 ± 78.21	195.33 ± 81.76	196.18 ± 86.16	190.25 ± 65.89	0.842
Penn classification (%)					0.917
Penn Class a	131.7 (77.9)	43.6 (77.9)	44.9 (80.1)	43.1 (75.8)	
Penn Class b	37.4 (22.1)	12.4 (22.1)	11.2 (19.9)	13.8 (24.2)	
Penn Class c	0.0 (0.0)	0.0 (0.0)	0.0 (0.0)	0.0 (0.0)	
Penn Class b and c	0.0 (0.0)	0 (0.0)	0.0 (0.0)	0.0 (0.0)	

*TAR+FET, total arch replacement with frozen elephant trunk; ABO, aortic balloon occlusion; HAR, hybrid aortic arch repair; MFS, Marfan syndrome; COPD, chronic obstructive pulmonary disease; CAD, coronary artery disease*.

### Operative Data

Table 3 shows the operative data among the 3 groups. The CPB time and ACC time were significantly longer in the ABO group than the other groups (*p* < 0.001). After MWM, the operation time was longer in the HAR group than the other groups (*p* = 0.039) (Table 4).

**Table 3:** Operative variables for the total cohort and each group before using the matching weight method.

	**Overall**	**HAR**	**TAR+FET**	**ABO**	***P*-value**
**Characteristic**	**(*n =* 633)**	**(*n =* 122)**	**(*n =* 431)**	**(*n =* 80)**	
Root Operation (%)					0.077
Bentall	128 (20.2)	14 (11.5)	98 (22.7)	16 (20.0)	
Root-sparing	480 (75.8)	99 (81.1)	319 (74.0)	62 (77.5)	
David	9 (1.4)	3 (2.5)	5 (1.2)	1 (1.2)	
Wheat's	16 (2.5)	6 (4.9)	9 (2.1)	1 (1.2)	
CABG (%)	77 (12.2)	20 (16.4)	46 (10.7)	11 (13.8)	0.209
Operation time (hour)	6.4 ± 1.9	6.5 ± 2.1	6.3 ± 1.9	6.5 ± 1.9	0.57
CPB (min)	163.8 ± 52.2	140.6 ± 58.5	165.7 ± 47.3	189.0 ± 53.7	<0.001
ACC (min)	101.1 ± 40.5	77.1 ± 34.8	103.4 ± 39.4	124.9 ± 36.9	<0.001

*TAR+FET, total arch replacement with frozen elephant trunk; ABO, aortic balloon occlusion; HAR, hybrid aortic arch repair; CABG, coronary artery bypass graft; CPB, cardiopulmonary bypass; ACC, aortic cross-clamp time*.

**Table 4:** Operative variables for the total cohort and each group after using the matching weight method.

	**Overall**	**HAR**	**TAR+FET**	**ABO**	***P*-value**
**Characteristic**	**(*n =* 169)**	**(*n =* 56.1)**	**(*n =* 56.1)**	**(*n =* 56.9)**	
Root operation (%)					0.072
Bentall	27.1 (16.0)	6.4 (11.3)	9.5 (17.0)	11.2 (19.7)	
Root-sparing	133.1 (78.7)	43.7 (77.9)	45.1 (80.4)	44.4 (77.9)	
David	3.2 (1.9)	2.3 (4.2)	0.5 (0.8)	0.4 (0.8)	
Wheat's	5.6 (3.3)	3.7 (6.6)	1.0 (1.8)	0.9 (1.6)	
CABG (%)	25.5 (15.1)	9.3 (16.6)	7.9 (14.2)	8.2 (14.5)	0.872
Operation time (hour)	6.47 ± 2.09	6.77 ± 2.41	6.11 ± 1.69	6.52 ± 2.07	0.039
CPB (min)	167.07 ± 53.93	144.66 ± 53.66	166.01 ± 46.56	190.18 ± 51.81	<0.001
ACC (min)	103.75 ± 39.48	80.33 ± 34.88	104.61 ± 34.92	125.96 ± 34.86	<0.001

*TAR+FET, total arch replacement with frozen elephant trunk; ABO, aortic balloon occlusion; HAR, hybrid aortic arch repair; CABG, coronary artery bypass graft; CPB, cardiopulmonary bypass; ACC, aortic cross-clamp time*.

### Clinical Outcomes Before MWM

Before MWM process, as shown in Table 5, the early outcomes were similar among the 3 groups.

**Table 5:** Early outcomes for the total cohort and each group before using the matching weight method.

	**Overall**	**HAR**	**TAR+FET**	**ABO**	***P*-value**
**Characteristic**	**(*n =* 633)**	**(*n =* 122)**	**(*n =* 431)**	**(*n =* 80)**	
Early mortality (%)	32 (5.1)	9 (7.4)	19 (4.4)	4 (5.0)	0.418
Stroke (%)	16 (2.5)	2 (1.6)	13 (3.0)	1 (1.2)	0.512
Paraplegia (%)	16 (2.5)	4 (3.3)	12 (2.8)	0 (0.0)	0.291
CRRT (%)	43 (6.8)	11 (9.0)	27 (6.3)	5 (6.2)	0.554
Re-exploration for bleeding (%)	24 (3.8)	5 (4.1)	15 (3.5)	4 (5.0)	0.792
Tracheotomy (%)	20 (3.2)	2 (1.6)	12 (2.8)	6 (7.5)	0.049
Hospital Stay (day)	13.9 ± 7.0	15.3 ± 8.8	13.6 ± 6.5	13.4 ± 6.6	0.048
ICU Stay (day)	7.7 ± 4.2	8.0 ± 4.2	7.5 ± 3.9	8.6 ± 5.7	0.052
Blood loss (ml)	846.0 ± 536.5	792.7 ± 349.3	875.9 ± 617.0	766.8 ± 166.3	0.117
RBC Transfusion Plasma Transfusion PLT Transfusion	4.1 ± 5.0 493.5 ± 538.0 3.0 ± 1.3	4.5 ± 3.1 449.6 ± 382.0 3.1 ± 1.4	4.0 ± 5.4 517.0 ± 585.7 3.0 ± 1.0	3.6 ± 5.6 433.8 ± 463.9 2.9 ± 1.9	0.447 0.27 0.523

*TAR+FET, total arch replacement with frozen elephant trunk; ABO, aortic balloon occlusion; HAR, hybrid aortic arch repair; CRRT, continuous renal replacement therapy*.

The overall 30-day mortality rate was 5.1%, and there was no statistical significance between the 3 groups with regard to 30-day mortality (HAR vs. TAR+FET vs. ABO: 7.4 vs. 4.4 vs. 5.0%, *p* = 0.418). There was no significant difference between the 3 groups with regard to stroke (HAR vs. TAR+FET vs. ABO: 1.6 vs. 3.0 vs. 1.2%, *p* = 0.512), paraplegia (HAR vs. TAR+FET vs. ABO: 3.3 vs. 2.8 vs. 0.0%, *p* = 0.291) and CRRT (HAR vs. TAR+FET vs. ABO: 9.0 vs. 6.3 vs. 6.2%, *p* = 0.554).

Our stroke rate was low. This might be explained by the younger age of the patients in our group. After statistical analyzing, the average age in our group was 51.3 ± 11.8 years, which was about 10 years younger compared to European and American reports (11). This was consistent with other Chinese reports on Chinese aortic dissection patients (12–14). For the younger patients, the atheromatous plaque was rarely found in the dissected aorta during the surgery.

### Clinical Outcomes After MWM

After MWM, there was no statistical significance between the 3 groups with regard to 30-day mortality. In addition, there was no statistically significant differences between the 3 groups with regard to stroke, paraplegia and re-exploration for bleeding, respectively. However, the tracheotomy rate was significantly higher in the ABO group (*p* = 0.001) (Table 6).

**Table 6:** Early outcomes for the total cohort and each group after using the matching weight method.

	**Overall**	**HAR**	**TAR+FET**	**ABO**	***P*-value**
**Characteristic**	**(*n =* 169)**	**(*n =* 56.1)**	**(*n =* 56.1)**	**(*n =* 56.9)**	
Early mortality (%)	11.2 (6.6)	4.5 (8.0)	3.4 (6.1)	3.3 (5.8)	0.783
Stroke (%)	5.0 (2.9)	2.2 (4.0)	1.8 (3.1)	1.0 (1.8)	0.679
Paraplegia (%)	4.0 (2.3)	1.9 (3.3)	2.1 (3.8)	0.0 (0.0)	0.104
CRRT (%)	11.5 (6.8)	3.6 (6.4)	4.4 (7.9)	3.5 (6.1)	0.834
Re-exploration for bleeding (%)	7.8 (4.6)	1.6 (2.8)	2.2 (3.9)	4.0 (7.0)	0.313
Tracheotomy (%)	7.6 (4.5)	0.9 (1.5)	1.4 (2.5)	5.4 (9.4)	0.001
Hospital Stay (day)	14.09 ± 7.48	14.87 ± 8.76	13.95 ± 7.22	13.46 ± 6.25	0.520
ICU Stay (day)	8.24 ± 4.88	7.60 ± 3.87	7.98 ± 4.18	9.14 ± 6.16	0.221
Blood loss (ml)	779.66 ± 350.21	759.85 ± 259.63	823.22 ± 524.48	756.28 ± 157.93	0.062
RBC Transfusion Plasma Transfusion PLT Transfusion	4.19 ± 5.35 481.36 ± 522.55 2.98 ± 1.63	4.53 ± 2.69 430.15 ± 344.68 3.05 ± 1.37	4.56 ± 7.26 552.32 ± 705.08 2.93 ± 1.11	3.48 ± 5.06 461.89 ± 446.70 2.97 ± 2.21	0.250 0.068 0.652

*TAR+FET, total arch replacement with frozen elephant trunk; ABO, aortic balloon occlusion; HAR, hybrid aortic arch repair; CRRT, continuous renal replacement therapy*.

The logistic regression analysis also showed no difference in early mortality between the 3 groups. (TAR+FET vs. HAR: OR = 0.75 (0.29,1.94), *P* = 0.554) (ABO vs. HAR: OR = 0.71 (0.19,2.70), *P* = 0.613).

## Comment

Surgery has been well-acknowledged to be the best selection for Debakey type I dissection. Presently, most published data only focused on the comparison between two techniques. Our research has compared the early outcomes of the three groups (Supplementary Figure 2). TAR+FET is an important operation for Debakey type I dissection especially when the tear was located in aortic arch. However, it was associated with inevitably the DHCA-related complications. Two other surgical techniques (HAR and ABO) have been employed to address this conundrum in our center. The HAR technique obviate the need for the circulatory arrest and the ABO technique shorten the circulatory arrest time to ~5 min. However, there is a paucity of data comparing these three surgical techniques, necessitating the current study.

In our study, we did not detect a significant difference between the TAR+FET, ABO, and HAR techniques with regard to 30-day mortality and post-operative adverse events. Convergent with our study, Hiraoka and colleagues (15) found no significant differences in 30-day, in-hospital, and operative mortality between HAR and TAR+FET groups. However, they found that the incidence of stroke in the HAR group was higher than the TAR+FET groups. The proportion of patients with ruptured aorta (*p* = 0.0302) was higher in the HAR group, which might cause atherosclerotic plaque instability, and the shedding of the carotid plaque might cause a higher stroke rate in the HAR group. The endograft delivery system, the rigid wires, or the bare-metal anchoring system may also be the potential risk factors for embolization (16, 17).

ABO technique was supposed to be superior to open total arch replacement with frozen elephant trunk theoretically. Previous study (18) showed that the ABO technique exerted some protective effects on the liver and kidney. It can also shorten the circulatory arrest time and elevate the lowest nasopharyngeal temperature to 28°C. The average ventilator supporting time was also shortened. However, the patients in the ABO group underwent longer ACC (126.6 ± 33.5 vs. 116.6 ± 40.9 min, *p* = 0.017) and CPB (193.5 ± 51.3 vs. 180.5 ± 57.9 min, *p* = 0.033) than the TAR+FET group in this study. It could be due the early stage of learning curve to this novel technique. We also found that the rate of tracheotomy was higher in the ABO group. The longer CPB duration might increase the risk of postoperative pulmonary infection, which leads to a high rate of tracheotomy.

In China, most of the patients underwent total arch replacement. Since Sun and his colleagues introduced this surgery in 2006 (2), TAR+FET has become increasingly popular in China. But it has not been widely adopted for type I aortic dissection throughout the world. Hemiarch or ascending aortic replacement yields favorable early outcomes, but they are associated with poor false lumen remodeling and high long-term reoperation rates (19). TAR+FET technique can effectively remove arch lesions, and promoting obliteration of the false lumen (1, 20). But it still carries a non-negligible risk of adverse events due to longer circulatory arrest. The hybrid technique does not necessitate circulatory arrest, but the patients in the HAR group are burdened with high surgical risk profile (21). Presently, we do not have a specific risk score to assist and evaluate the patients who underwent aortic surgery (22). HAR may be a better option for patients with preoperative liver and kidney insufficiency or organ malperfusion (19). But the stroke rate and the number of late aortic events was higher in the HAR group (compared with TAR+FET). And it was also associated with reduced long-term survival beyond 5 years (23). So HAR technique is suitable for patients with significant comorbidities. ABO technique also has protective effects on liver and kidney (18). It may be a better option for patients with hepatorenal dysfunction, combining with high risk of stroke. But it had the longest ACC and CPB among the three groups according to our present findings. As a relatively novel technique, it needs more robust evidence. The key difference between these 3 techniques relies on the HCA time. Some researchers propose that the shorter the circulatory arrest take, the better the patients become. But rapid temperature changes may lead to brain tissue damage. More long-term clinical studies investigations are warranted to determine which surgical strategy yield superior clinical outcome, and whether they remain to be of equivalent efficacy and safeties.

## Limitations

As a retrospective study, our study also had some limitations. The number of patients in the ABO group was limited and we can only explore the early outcomes. Because the ABO technique is a relatively novel surgery, which was first performed in 2016. ABO technique is a relatively novel surgery, which was first performed in 2017. We are now enrolling more patients undergoing ABO surgery into the registry. Second, three kinds of techniques were performed by different groups in our institute. However, the surgeries were performed by experienced surgeons who were selected by rigid standards in our center.

## Conclusion

Based on the early outcomes, the three TAR approaches were equally applicable to type I aortic dissection. We may choose the specific procedure relatively flexibly according to patient status and surgeon's expertise. Importantly, long-term investigations are warranted to determine whether they remain to be of equivalent efficacy and safety.

## Data Availability Statement

The raw data supporting the conclusions of this article will be made available by the authors, without undue reservation.

## Ethics Statement

This study was approved by the Ethics Committee of Fuwai Hospital (Approval NO:2017-877).

## Author Contributions

JW, JuQ, EX, and CY developed study design. EX wrote the paper. JW, JuQ, EX, LD, JiQ, QL, WJ, RZ, SF, FC, WG, HG, XS, and CY organized patient recruitment and collected study statistics. EX and CY applied for the funding. JuQ, EX, and JW were involved in the statistical analyses and diagramming. All authors contributed to the article and approved the submitted version.

## Conflict of Interest

The authors declare that the research was conducted in the absence of any commercial or financial relationships that could be construed as a potential conflict of interest.

## Abbreviations

ACP: antegrade cerebral perfusion
TAR: total arch replacement
FET: frozen elephant trunk
ABO: aortic balloon occlusion
HAR: hybrid aortic arch repair
DHCA: deep hypothermic circulatory arrest
CPB: cardiopulmonary bypass time
ACC: aortic cross-clamp time
SMD: standardized mean difference
CRRT: continuous renal replacement therapy
OR: odds ratio
MWM: matching weight method
IPTW: inverse probability of treatment weighting.
