# Supplementary material for: Early Outcomes of Three Total Arch Replacement Strategies for DeBakey Type I Aortic Dissection
Source: Front Cardiovasc Med. 2021 Apr 15;8:638420. doi: 10.3389/fcvm.2021.638420 (PMC8081908; doi:10.3389/fcvm.2021.638420)
Supplement: Supplementary file 1 [file Presentation_1.pdf]

Supplementary Material

Early outcomes of three total arch replacement strategies for DeBakey type I aortic dissection

Enzehua Xie

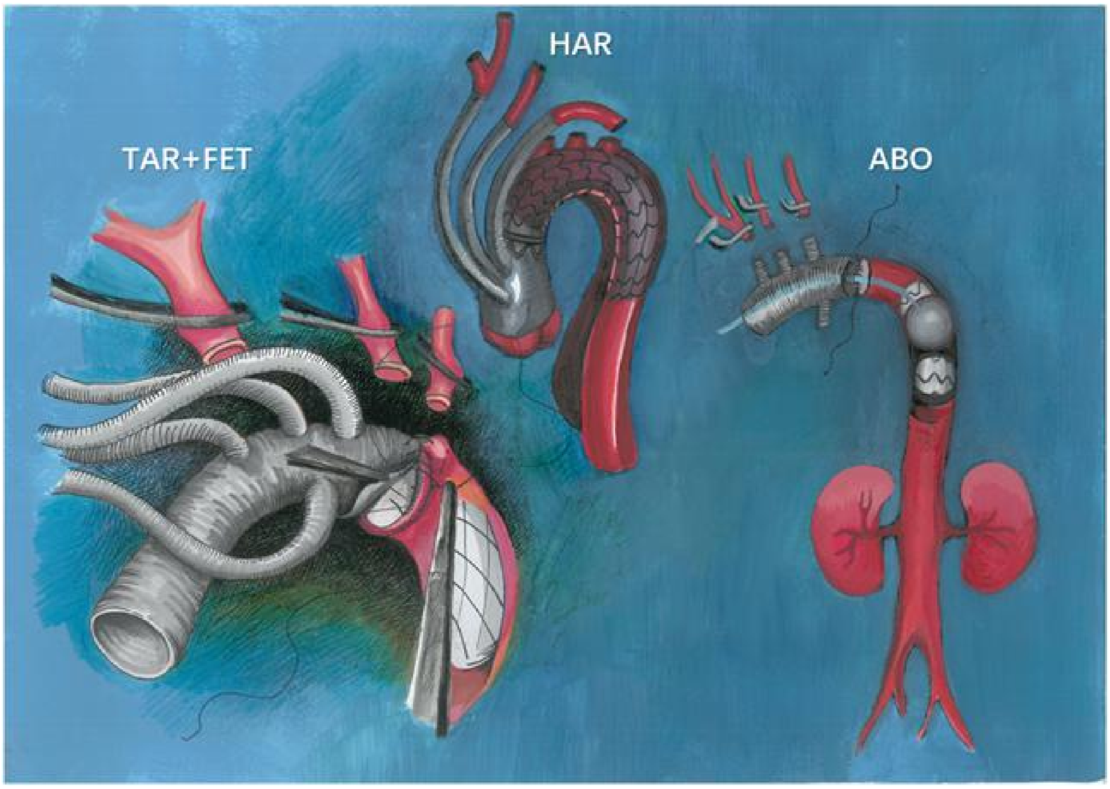

Supplementary Figure 1. Three total arch replacement strategies.

TAR+FET, total arch replacement with frozen elephant trunk; ABO, aortic balloon occlusion; HAR, hybrid aortic arch repair.

Three total arch replacement strategies in DeBakey type I dissection

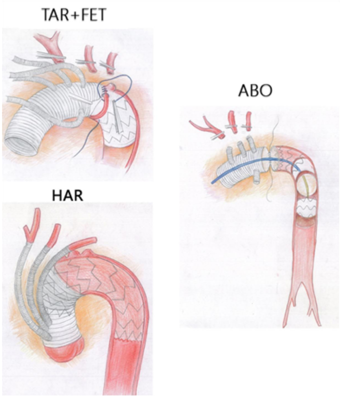

Patient enrollment
2016/1/1-2018/12/31
Fuwai Hospital
DeBakey type I dissection

Total cases: 633

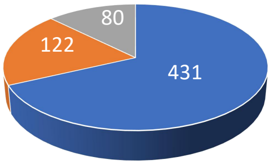

TAR+FET HAR ABO

Before MWM

	HAR	TAR+FET	ABO	P
Early mortality(%)	9 (7.4)	19 (4.4)	4 (5.0)	0.418
Stroke (%)	2 (1.6)	13 (3.0)	1 (1.2)	0.512
Paraplegia (%)	4 (3.3)	12 (2.8)	0 (0.0)	0.291
CRRT (%)	11 (9.0)	27 (6.3)	5 (6.2)	0.554

After MWM

	HAR	TAR+FET	ABO	P
Early mortality (%)	4.5 (8.0)	3.4 (6.1)	3.3 (5.8)	0.783
Stroke (%)	2.2 (4.0)	1.8 (3.1)	1.0 (1.8)	0.679
Paraplegia (%)	1.9 (3.3)	2.1 (3.8)	0.0 (0.0)	0.104
CRRT (%)	3.6 (6.4)	4.4 (7.9)	3.5 (6.1)	0.834

Similar early outcomes were achieved among patients undergoing TAR+FET, ABO and HAR repair.

Supplementary Figure 2. Schematic illustration of this article.

TAR+FET, total arch replacement with frozen elephant trunk; ABO, aortic balloon occlusion; HAR, hybrid aortic arch repair.
